# Circulating mir-199-3p screens the onset of type 2 diabetes mellitus and the complication of coronary heart disease and predicts the occurrence of major adverse cardiovascular events

**DOI:** 10.1186/s12872-023-03601-4

**Published:** 2023-11-16

**Authors:** Renjie Ruan, Yanwei Liu, Xiang Zhang

**Affiliations:** 1 Department of Cardiology, The Third Affiliated Hospital of Wenzhou Medical University, Wenzhou, 325000 China; 2 Department of Emergency, The Affiliated Hospital of Qingdao University, Qingdao, 266003 China; 3 Department of Cardiology, People’s Hospital of Rizhao, No.126 Taian Road, Donggang District, Rizhao, 276827 China

**Keywords:** Complications, Cardiovascular Disease, miRNA, Biomarker, Prognosis

## Abstract

**Background:**

Coronary heart disease (CHD) is a major complication of type 2 diabetes mellitus (T2DM), which causes an adverse prognosis. There is an urgent need to explore effective biomarkers to evaluate the patients’ adverse outcomes.

**Objective:**

This study aimed to identify a novel indicator for screening T2DM and T2DM-CHD and predicting adverse prognosis.

**Materials and methods:**

The study enrolled 52 healthy individuals, 85 T2DM patients, and 97 T2DM patients combined with CHD. Serum miR-199-3p levels in all study subjects were detected with PCR, and its diagnostic significance was evaluated by receiver operating curve (ROC) analysis. The involvement of miR-199-3p in disease development was assessed by the Chi-square test, and the logistic regression analysis was performed to estimate the risk factor for major adverse cardiovascular events (MACE) in T2DM-CHD patients.

**Results:**

Significant downregulation of miR-199-3p was observed in the serum of both T2DM and T2DM-CHD patients, which discriminated patients from healthy individuals and distinguished T2DM and T2DM-CHD patients. Reduced serum miR-199-3p was associated with the increasing blood glucose, glycated hemoglobin (HbA1c), and homeostasis model assessment-insulin resistance index (HOMA-IR) of T2DM patients and the increasing triglycerides (TG), low-density lipoprotein (LDL), fibrinogen, and total cholesterol (TC) and decreasing high-density lipoprotein (HDL) of T2DM-CHD patients. miR-199-3p was also identified as a biomarker predicting the occurrence of MACE.

**Conclusion:**

Downregulated miR-199-3p could screen the onset of T2DM and its complication with CHD. Reduced serum miR-199-3p was associated with the severe development of T2DM and T2DM-CHD and predicted the adverse outcomes of T2DM-CHD patients.

**Supplementary Information:**

The online version contains supplementary material available at 10.1186/s12872-023-03601-4.

## Introduction

Type II diabetes mellitus (T2DM) is a common chronic metabolic disease, which has been considered a major risk factor for cardiovascular diseases. Long-term hyperglycemia promotes atherosclerosis, exacerbates endometrial damage and oxidative stress, accelerates inflammation, and therefore leads to coronary artery injury [1–3]. Coronary heart disease (CHD) is a common complication of T2DM, and the number of new cases of T2DM combined with CHD has gradually increased in the past decades. The disease conditions of T2DM patients combined with CHD are more complex, which promotes disease development and therefore increases the difficulty of therapy [4]. Although great progress has been made in the treatment of T2DM combined with CHD, exploring effective biomarkers and risk factors is still of great significance in improving its targeted therapy [5].

Thanks to the development of molecular biology, the identification of disease biomarkers has advanced in recent years, where the significance of non-coding RNAs (ncRNAs) has attracted huge attention [6–8]. The dysregulation of miRNAs would regulate the process of atherosclerosis, including endothelial dysfunction, endothelium mesenchymal transformation, macrophage activation, vascular smooth muscle cell proliferation and motility, platelet hyperactivity, and calcification [9–11]. In previous studies, there have been several reports exploring functional microRNAs (miRNAs) that mediated the progression of T2DM and CHD [12–15]. Due to the inconsistency of miRNA expression in different diseases, some miRNAs showed opposite expression trends in T2DM and CHD. In a recent study, differentially expressed miRNAs in T2DM were identified and constructed co-expression networks, which were considered biomarker candidates of T2DM. Among the dysregulated miRNAs, miR-199-3p showed a significantly reduced expression [16]. The downregulation of miR-199-3p in the urine of diabetes mellitus (DM) patients was reported to act as a diagnostic biomarker that discriminated against DM patients and predicted the nephropathy of patients [17]. The abnormal expression of miR-199-3p was previously reported to regulate inflammation and mediate disease development of allergic rhinitis, hepatocellular carcinoma, and diabetic nephropathy [17–20]. Therefore, reduced miR-199-3p was also speculated to be involved in the development of T2DM patients with CHD, which was confirmed in the present study. Additionally, major adverse cardiovascular events (MACE), defined as non-fatal stroke, non-fatal myocardial infarction, and cardiovascular death, are one of the main adverse prognostic factors for T2DM patients with CHD [21, 22]. Therefore, predicting the occurrence of MACE is critical to timely adjust therapeutic strategies and improve patients’ outcomes. Hence, except for evaluating the significance of miR-199-3p in disease severity and aggravation, its function in assessing the risk of MACE in T2DM patients combined with CHD was also investigated.

## Materials and methods

### Study subjects and profiles

There were three groups of patients included in this study. The healthy group included 52 individuals who received routine physical examination in our hospital and the results showed no abnormality. The T2DM group enrolled 85 T2DM patients diagnosed according to the American Diabetes Association guidelines [23, 24]. Patients with unstable angina pectoris, positive non-invasive cardiac function results, cerebrovascular disease, coronary artery disease, peripheral artery disease, and acute coronary syndrome were excluded from the T2DM group. The T2DM-CHD group included 97 T2DM patients diagnosed with CHD. Patients with a history of CHD, or diagnosed with type 1 diabetes mellitus, stroke, myocardial infarction, malignant tumors, liver and kidney dysfunction, chronic inflammation, and autoimmune diseases were excluded. All participants were enrolled at the People’s Hospital of Rizhao.

This study had been approved by the Ethics Committee of the People’s Hospital of Rizhao. All study subjects had signed informed consent and the clinical records of T2DM and T2DM-CHD groups were completed. The clinicopathological information was collected after their diagnosis and a 1-year follow-up survey was performed through telephone or outpatient review to monitor the occurrence of MACE and patients’ outcomes. The MACE in the present study was defined as all-cause deaths, non-fatal myocardial infarction, target lesion revascularization, recurrent angina pectoris, and acute heart failure. For patients with one of the above items, the follow-up was completed.

### Biochemical tests

The serum indicators, including total cholesterol (TC), triglyceride (TG), low-density lipoprotein (LDL), and high-density lipoprotein (HDL) were detected by a full automatic biochemical analyzer (Hitachi, Japan). The fasting blood glucose (FBG) was measured with a HemoCue glucose analyzer, and the homeostasis model assessment- insulin resistance index (HOMA-IR) was calculated with the equation: HOMA-IR = (FBG × insulin)/405. The glycated hemoglobin (HbA1c) was measured with the boronate affinity high-performance liquid chromatography method. Fibrinogen was analyzed using the BNII Nephelometer 100 Analyzer (Dade Behring, USA).

### Sample collection and real-time quantitative PCR

Fasting venous blood samples were collected from all participants in the morning after the enrollment. Serum was obtained by centrifugation at 3000 r/min for 10 min and stored at -80 °C.

Samples were lysed with Trizol reagent (Invitrogen, USA) to isolate total RNA. cDNA was generated by reverse transcription using TaqMan miRNA Reverse Transcription Kit (Applied Biosystem, USA) and amplified on the 7500 Real-time PCR system (Applied Biosystem, USA) using SYBR Green kit (Tiangen, China). The relative expression of miR-199-3p was calculated using the 2^−ΔΔCT^ method normalized to cel-miR-39. The primer sequences of miR-199-3p and cel-miR-39 were: miR-199-3p forward 5’- ACACTCCAGCTGGGTCCCTGAGACCCTTTA-3’, miR-199-3p reverse 5’-CTCAACTGGTGTCGTGGAGTCGGCAATTCA-3’; cel-miR-39 forward 5’-UCACCGGGUGUAAAUCAGCUUG-3’, reverse: 5’-TCACCGGGTGTAAAT CAGCTTG-3’.

### Cell culture and treatments

Human peripheral blood mononuclear cells (hPBMCs) from healthy and T2DM patients were purchased from Precision for Medicine and incubated in RPMI culture medium with 10% FBS at 37 °C.

Human cardiomyocyte, AC16 cell was obtained from Shanghai Meiyan Biological Technology Co. and maintained in the DMEM culture medium with 10% FBS at 37 °C. AC16 cells were treated with 30 mmol/L glucose to mimic cardiomyocyte injury during T2DM.

### Cell transfection

Glucose-treated AC16 cells were transfected with miR-199-3p mimic, CD151 overexpression plasmid, or their negative controls using Lipofectamine 2000 (Invitrogen, USA) at room temperature. Successful transfection was confirmed by the expression of miR-199-3p and CD151 using real-time qPCR described as above.

### Dual-luciferase reporter assay

The binding sites between miR-199-3p and CD151 were predicted from online database (https://rnasysu.com/encori/index.php). Wild-type and mutant-type plasmids were constructed by cloning wild-type binding sites and mutant sites into pGL3 vectors. Glucose-treated AC16 cells were co-transfected with established plasmids and miR-199-3p mimic, inhibitor, or negative control using Lipofectamine 2000. The luciferase activity of CD151 was detected by the luciferase reporter assay system normalized to Renilla.

### Statistical analyses

Data were expressed as mean ± SD. (n = the number of study subjects in each group). Difference comparison was performed with one-way ANOVA (*P* < 0.05). The ROC analysis was employed to evaluate the significance of miR-199-3p discriminating T2DM patients with CHD (AUC > 0.5 indicates the significant discriminating value). The logistic regression analysis was used to explore the risk factors for the occurrence of MACE in T2DM patients with CHD.

## Results

### Comparison of baseline information

The enrolled subjects possessed matched age and gender composition with no significant differences. T2DM patients always lose weight, and the BMI of T2DM and T2DM-CHD patients was lower than healthy individuals, but the difference was not significant (Table 1). T2DM and T2DM-CHD patients showed higher levels of FBG, TG, LDL, HbA1c, fibrinogen, TC, and HOMA-IR and a lower level of HDL. Moreover, T2DM-CHD patients showed significantly increasing levels of TG, LDL, HbA1c, fibrinogen, TC, and a decreasing level of HDL compared with T2DM patients (Table 1).

**Table 1:** Baseline information of study subjects

	Healthy individuals	T2DM patients	T2DM-CHD patients	*P*-value
Age (years)	53.38 ± 8.59	52.75 ± 9.66	53.02 ± 8.38	0.922
Gender (M, %)	33/19	54/31	58/39	0.847
BMI (kg/m^2^)	24.43 ± 3.53	23.09 ± 2.51	23.50 ± 2.91	0.054
FBG (mmol/L)	4.79 ± 1.39	7.99 ± 18.83	8.19 ± 1.65	< 0.001
TG (mmol/L)	1.41 ± 0.23	1.68 ± 0.36	2.27 ± 0.29	< 0.001
HDL (mmol/L)	1.41 ± 0.26	1.13 ± 0.24	0.83 ± 0.12	< 0.001
LDL (mmol/L)	2.34 ± 0.59	2.77 ± 0.40	3.07 ± 0.32	< 0.001
HbA1c (%)	4.99 ± 0.41	7.82 ± 0.72	8.28 ± 0.44	< 0.001
Fibrinogen (mg/dL)	90.85 ± 8.40	194.45 ± 9.15	212.78 ± 7.11	< 0.001
TC (mmol/L)	3.83 ± 0.41	4.66 ± 0.56	5.49 ± 0.36	< 0.001
HOMA-IR	0.80 ± 0.10	2.68 ± 0.47	2.87 ± 0.34	< 0.001

BMI: body mass index; FBG: fasting blood glucose; TG: triglyceride; HDL: high-density lipoprotein; LDL: low-density lipoprotein; HbA1c: glycated hemoglobin; TC: total cholesterol; HOMA-IR: homeostasis model assessment- insulin resistance index

### Expression of mir-199-3p in T2DM and T2DM-CHD and its diagnostic significance

**Fig. 1:**
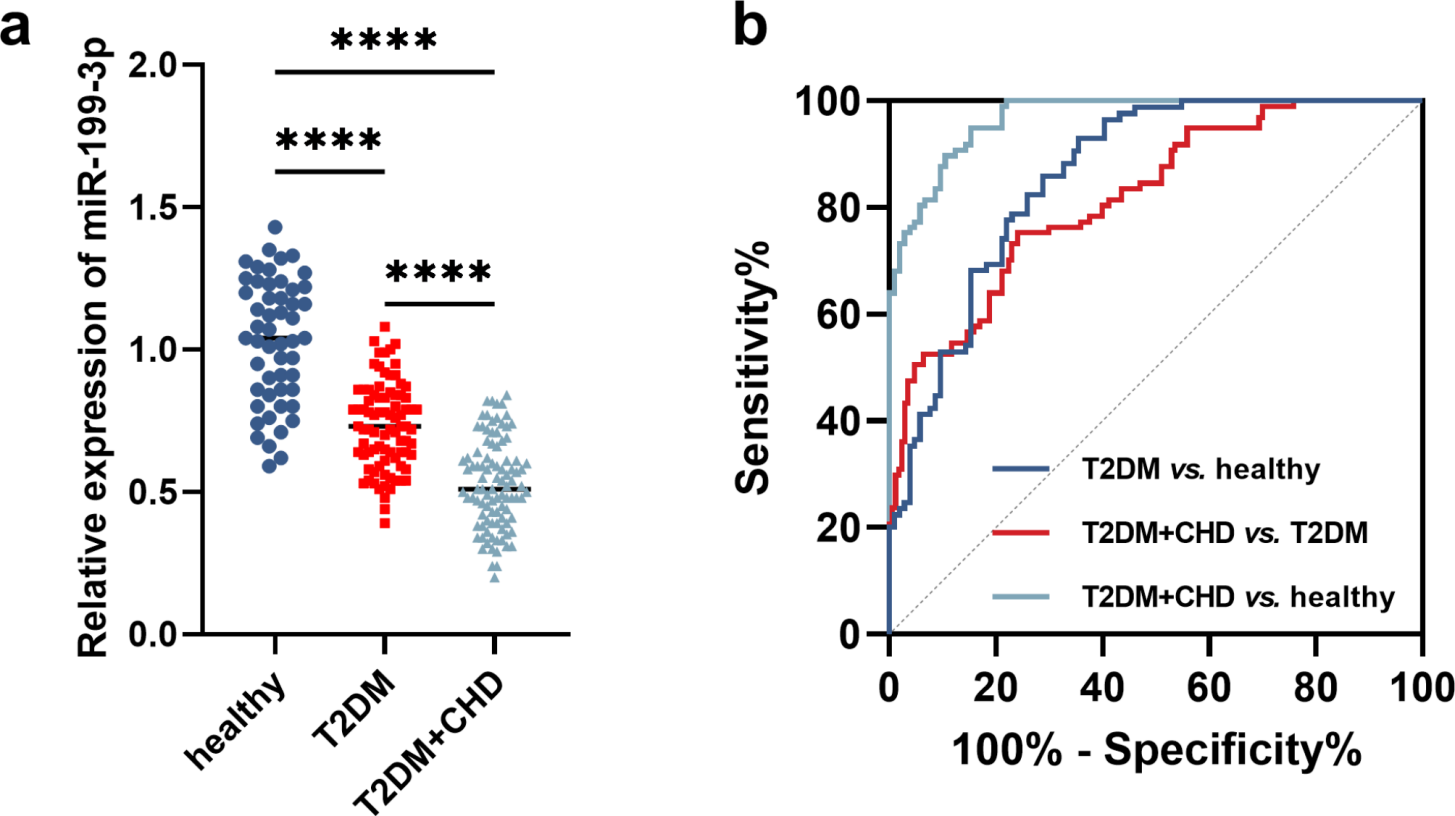
(**a**) miR-199-3p was downregulated in the serum of T2DM and T2DM-CHD patients compared with healthy individuals, and the difference between T2DM and T2DM-CHD patients was significant. ****P < 0.0001. (**b**) ROC curve based on miR-199-3p expression. miR-199-2p could discriminate T2DM and T2DM-CHD patients from healthy individuals and distinguish between T2DM and T2DM-CHD patients

In T2DM and T2DM-CHD patients, serum miR-199-3p was significantly downregulated relative to healthy individuals, and T2DM-CHD patients showed a much lower miR-199-3p level than in T2DM patients (Fig. 1a). The downregulation of miR-199-3p was also observed in hPBMCs from T2DM patients compared with healthy hPBMCs (Figure S1). With the employment of ROC, the reduced serum miR-199-3p was found to discriminate T2DM (area under the curve, AUC = 0.860, sensitivity = 85.88%, specificity = 71.15%) and T2DM-CHD (AUC = 0.968, sensitivity = 94.85%, specificity = 84.62%) patients from healthy individuals. Additionally, the lower miR-199-3p in T2DM-CHD patients could also discriminate patients from T2DM patients with the AUC of 0.819 (sensitivity = 75.26%, specificity = 75.29%, Fig. 1b).

### Significance of mir-199-3p in the development of T2DM and T2DM-CHD

According to the average serum miR-199-3p (0.731), T2DM patients were divided into a low-miR-199-3p group and a high-miR-199-3p group. The low-miR-199-3p group included 45 patients composed of 29 males and 16 females, while the high-miR-199-2p group included 40 patients composed of 25 males and 15 females. Patients in the low-miR-199-3p group showed higher FBG (*P* < 0.001), HbA1c (*P* < 0.001), fibrinogen (*P* = 0.041), and HOMA-IR (*P* = 0.038), and significant associations were confirmed (Table 2).

**Table 2:** Correlation of miR-199-3p with T2DM patients’ clinicopathological features

	Cases (n = 85)	Low-miR-199-3p	High-miR-199-3p	*P*-value
Age				0.657
< 55	51	26	25	
≥ 55	34	19	15	
Gender				0.853
Male	54	29	25	
Female	31	16	15	
BMI				0.486
< 23	37	18	19	
≥ 23	48	27	21	
FBG				< 0.001
< 8.0	44	15	29	
≥ 8.0	41	30	11	
TG				0.229
< 1.7	43	20	23	
≥ 1.7	42	25	17	
HDL				0.495
< 1.2	52	26	26	
≥ 1.2	33	19	14	
LDL				0.411
< 2.8	47	23	24	
≥ 2.8	38	22	16	
HbA1c				< 0.001
< 7.8	37	9	28	
≥ 7.8	48	36	12	
Fibrinogen				0.041
< 195	41	17	24	
≥ 195	44	28	16	
TC				0.443
< 4.7	43	21	22	
≥ 4.7	42	24	18	
HOMA-IR				0.038
< 2.7	43	18	25	
≥ 2.7	42	27	15	

BMI: body mass index; FBG: fasting blood glucose; TG: triglyceride; HDL: high-density lipoprotein; LDL: low-density lipoprotein; HbA1c: glycated hemoglobin; TC: total cholesterol; HOMA-IR: homeostasis model assessment- insulin resistance index

For T2DM-CHD patients, patients were also grouped according to the mean value of serum miR-199-3p (0.529). miR-199-3p was significantly related with the FBG (*P* < 0.001), TG (*P* = 0.034), HDL (*P* = 0.020), LDL (*P* = 0.048), HbA1c (*P* = 0.019), fibrinogen (*P* = 0.020), TC (*P* = 0.006), and HOMA-IR (*P* = 0.019) of patients (Table 3), indicating the potential involvement of miR-199-3p in the development of T2DM and T2DM-CHD.

**Table 3:** Correlation of miR-199-3p with T2DM-CHD patients’ clinicopathological features

	Cases (n = 97)	Low-miR-199-3p	High-miR-199-3p	*P*-value
Age				0.886
< 55	55	28	27	
≥ 55	42	22	20	
Gender				0.432
Male	58	28	30	
Female	39	22	17	
BMI				0.384
< 23	39	18	21	
≥ 23	58	32	26	
FBG				< 0.001
< 8.0	44	14	30	
≥ 8.0	53	36	17	
TG				0.034
< 2.3	47	19	28	
≥ 2.3	50	31	19	
HDL				0.020
< 0.8	38	14	24	
≥ 0.8	59	36	23	
LDL				0.048
< 3.1	54	23	31	
≥ 3.1	43	27	16	
HbA1c				0.019
< 8.3	50	20	30	
≥ 8.3	47	30	17	
Fibrinogen				0.020
< 212	46	18	28	
≥ 212	51	32	19	
TC				0.006
< 5.5	50	19	31	
≥ 5.5	47	31	16	
HOMA-IR				0.019
< 2.9	50	20	30	
≥ 2.9	47	30	17	

BMI: body mass index; FBG: fasting blood glucose; TG: triglyceride; HDL: high-density lipoprotein; LDL: low-density lipoprotein; HbA1c: glycated hemoglobin; TC: total cholesterol; HOMA-IR: homeostasis model assessment- insulin resistance index

### Significance of mir-199-3p in predicting the occurrence of MACE in T2DM-CHD patients

**Fig. 2:**
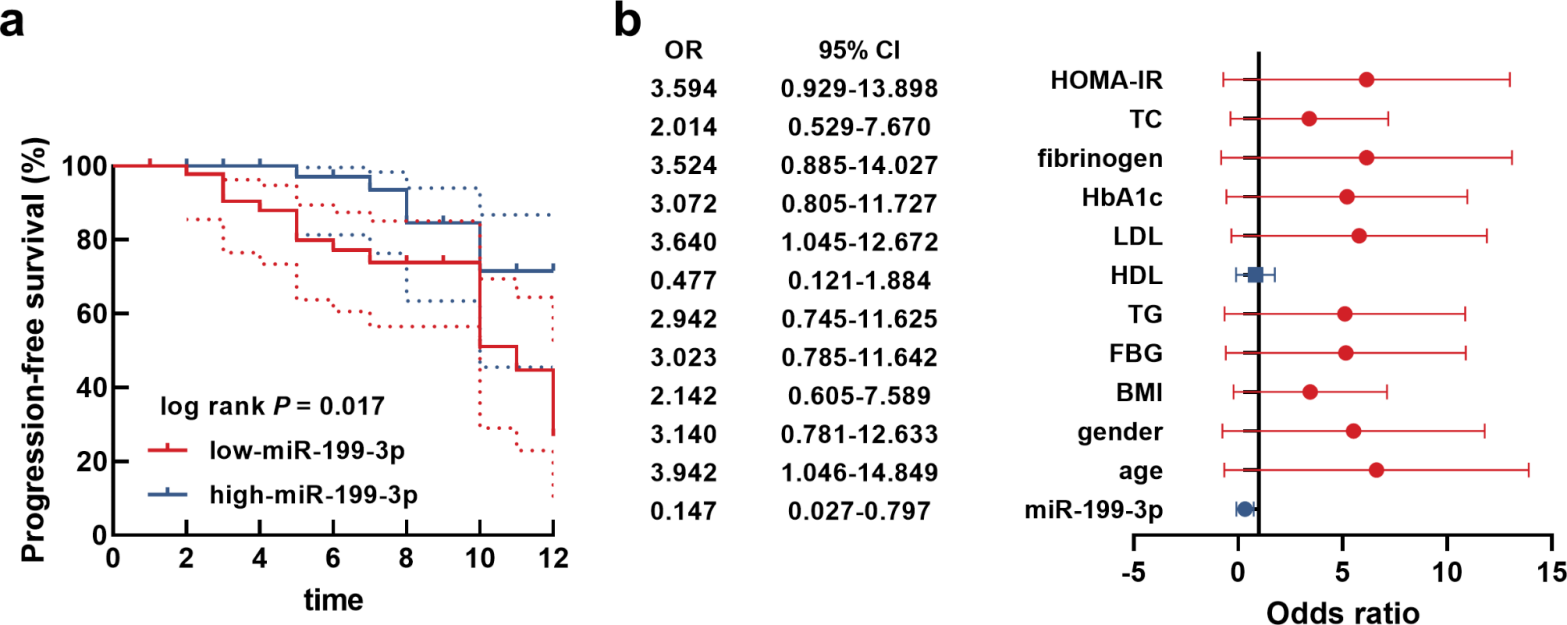
(**a**) Lower serum miR-199-3p was closely associated with the progression-free survival of T2DM-CHD patients. The endpoint events were defined as the occurrence of MACE, including all-cause deaths, non-fatal myocardial infarction, target lesion revascularization, recurrent angina pectoris, and acute heart failure. (**b**) logistic regression analysis identified miR-199-3p, age, and LDL as risk factors for the occurrence of MACE

The occurrence of MACE in T2DM-CHD patients was summarized by a 1-year follow-up survey. It was found that patients in the low-miR-199-3p group were easier to occur MACE than patients in the high-miR-199-3p (Fig. 2a). Moreover, miR-199-3p was also identified as a risk factor for the occurrence of MACE in T2DM-CHD patients with the OR of 0.147 (*P* = 0.026), as well as the age (odds ratio, OR = 3.942, *P* = 0.043) and LDL (OR = 3.640, *P* = 0.042) of patients (Fig. 2b).

### Potential regulatory mechanism underlying mir-199-3p in cardiomyocyte

**Fig. 3:**
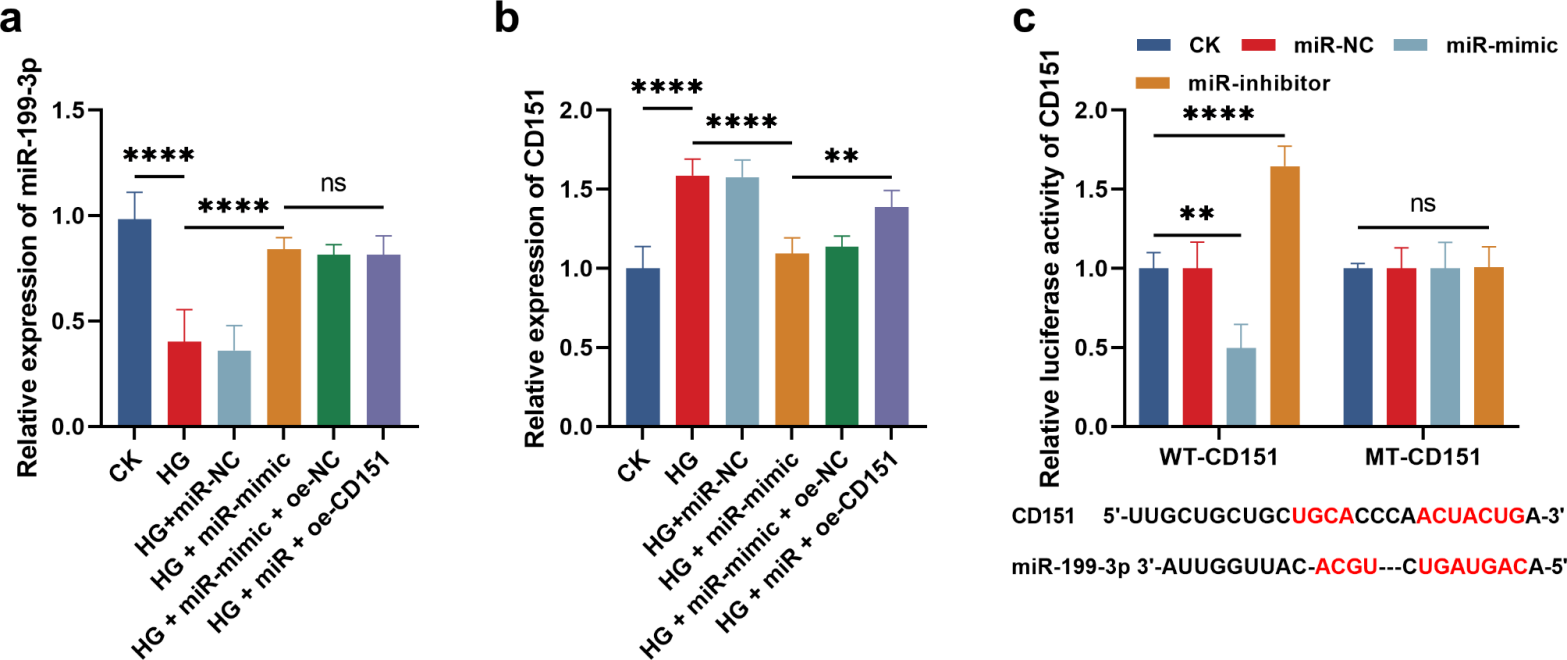
(**a**-**b**) Glucose treatment induced reduced miR-199-3p (**a**) and increased CD151 (**b**) in cardiomyocytes. Overexpressing miR-199-3p significantly suppressed the expression of CD151 in glucose-treated cardiomyocytes, which was reversed by the transfection of CD151 overexpression plasmid. c. miR-199-3p could bound with the 3’UTR of CD151 and negatively regulate the luciferase activity of CD151 in glucose-treated cardiomyocytes. ^ns^*P* > 0.005, **P < 0.01, ****P < 0.0001

Compared with untreated cardiomyocytes, miR-199-3p was significantly downregulated in glucose-treated AC16 cells (Fig. 3a), and increased CD151 was also observed (Fig. 3b). miR-199-3p overexpressing cells were successfully established by the transfection of miR-199-3p mimic (Fig. 3a), which showed significantly inhibitory effect on CD151 (Fig. 3b). Consistently, the overexpression of miR-199-3p dramatically suppressed the luciferase activity of CD151, while its knockdown showed opposite effects (Fig. 3c).

## Discussion

Abnormal metabolism of glucose increases the incidence of cardiovascular diseases, and T2DM has been identified as an independent risk factor for the onset of CHD [25–27]. Additionally, due to the intersected pathogenic mechanism of T2DM and CHD, CHD has also become one of the most common complications and causes of death of T2DM. The risk factors for combining CHD with T2DM have been widely identified in previous studies, such as aging, hypertension, and abnormal metabolism of lipids [28]. However, the identification of biomarkers predicting the development and outcomes of T2DM patients with CHD has attracted minor attention. The significance of miRNAs, especially the dysregulated miRNAs in human diseases, has been widely confirmed. Both T2DM and CHD were considered metabolic diseases [29]. miR-199-3p were previously identified as biomarker candidates in gestational diabetes and diabetic nephropathy and were indirectly correlated with the levels of HDL and TG [17, 30, 31], indicating its significance in metabolic diseases. Moreover, miR-199-3p was reported to be associated with the calcification of the aortic valve and mediate the enrichment of cardiomyocyte and sinus nodal cells [32, 33]. Therefore, miR-199-3p was speculated to participate in the development of T2DM and the occurrence of CHD. In the present study, we observed the downregulation of miR-199-3p in the serum of T2DM patients and T2DM patients with CHD, which could discriminate T2DM and T2DM-CHD patients from healthy individuals. The reduced serum miR-199-3p could also distinguish T2DM and T2DM-CHD patients, suggesting its potential in predicting the occurrence of CHD in T2DM patients.

On the other hand, decreasing miR-199-3p was found to be correlated with the increasing blood glucose, HbA1c, and HOMA-IR of T2DM patients. HbA1c and HOMA-IR have been employed in the clinic to assess the severity of diabetes indicating the conditions of glycemic control and insulin resistance [34–37]. For T2DM-CHD patients, except for the T2DM indicators, lower serum miR-199-3p was related with the increasing TG, LDL, fibrinogen, and TC and decreasing HDL of patients. The disturbed lipid metabolism in T2DM patients led to abnormal levels of blood lipid indexes, increasing the risk of CHD. Fibrinogen has also been demonstrated to be closely correlated with the pathogen and progression of CHD through the mechanism of stimulating smooth muscle cell growth, promoting atherosclerosis, mediating thrombogenesis, and regulating hemorheology [38]. Fibrinogen has also been identified as an indicator of the severity and prognosis of coronary artery disease [39, 40]. A recent study also confirmed that fibrinogen is correlated with the 5-year prognosis of T2DM patients with coronary artery disease who received percutaneous coronary therapy [41]. Hence, miR-199-3p was also involved in the development of T2DM-CHD patients. However, the expression of miR-199-3p was not traced during disease development, which would further confirm its involvement. There was a lack of control of CHD patients to verify the differential expression of miR-199-3p in T2DM-CHD, which would help clear the specificity of miR-199-3p in T2DM-induced CHD. Further investigation should expand to include CHD patients.

MACE is the main manifestation of poor prognosis in CHD patients. In this study, all-cause deaths, non-fatal myocardial infarction, target lesion revascularization, recurrent angina pectoris, and acute heart failure were observed in T2DM-CHD patients. In previous studies, several circulating miRNAs have been revealed to predict MACE in various human diseases. For instance, miR-411-5p was reported to serve as a prognostic biomarker predicting MACE in atrial fibrillation patients [42]. Serum miR-497-5p was revealed to screen the onset of acute coronary syndrome and indicate the occurrence of MACE after percutaneous coronary intervention [43]. Herein, the lower serum miR-199-3p level was significantly associated with the high occurrence of MACE and miR-199-3p was identified as a risk factor for MACE in T2DM-CHD patients. However, due to the individualized treatments of patients, there might be differences in the outcomes of patients, and the prognostic significance of miR-199-3p should be specified under different interventions. Moreover, the mechanism underlying the predictive value of miR-199-3p needs further exploration.

To evaluate the potential regulatory mechanism underlying miR-199-3p, the downstream miRNA of miR-199-3p was predicted and evaluated in glucose-treated cardiomyocyte mimicking cardiomyocyte injury during T2DM. A recent study on cardiac development and regeneration noticed the regulatory effect of miR-199-3p, where CD151 was revealed to mediate the function of miR-199-3p [44]. Here, CD151 was found to be upregulated in glucose-treated cardiomyocyte, which was negatively regulated by miR-199-3p. Therefore, miR-199-3p was hypothesized to regulate the development of CHD induced by T2DM via modulating CD151, which needs further confirmation. In addition, the abnormal expression of miR-199-3p and specific function needs in vivo validation by animal models. Rat and mice are commonly used animals for modeling, but mammalian species would be closer to the disease pathogen and progression in the human. Hence, mammalian animal modeling should be considered in future research.

In conclusion, downregulated miR-199-3p screened the pathogen of T2DM and its complication of CHD. Reduced serum miR-199-3p was significantly associated with the severity and progression of T2DM and T2DM-CHD patients and served as a risk factor for MACE predicting the adverse prognosis of T2DM-CHD patients. miR-199-3p negatively regulated CD151, which might be the regulatory mechanism underlying its involvement in T2DM-CHD development. The miR-199-3p/CD151 axis provides a potential therapeutic target for T2DM and its complication with CHD. The sensitivity of the miR-199-3p/CD151 axis to drugs should be assessed in the future studies, which would benefit the development of targeted therapies for T2DM and T2DM-CHD.

### Electronic supplementary material

Below is the link to the electronic supplementary material.

Supplementary Material 1

## Data Availability

Corresponding authors may provide data and materials.

## List of abbreviations

CHD: Coronary heart disease
MACE: Major adverse cardiovascular events
T2DM: Type II diabetes mellitus
