# Supplementary figures and images for: Circulating mir-199-3p screens the onset of type 2 diabetes mellitus and the complication of coronary heart disease and predicts the occurrence of major adverse cardiovascular events
Source: BMC Cardiovasc Disord. 2023 Nov 16;23:563. doi: 10.1186/s12872-023-03601-4 (PMC10655316; doi:10.1186/s12872-023-03601-4)

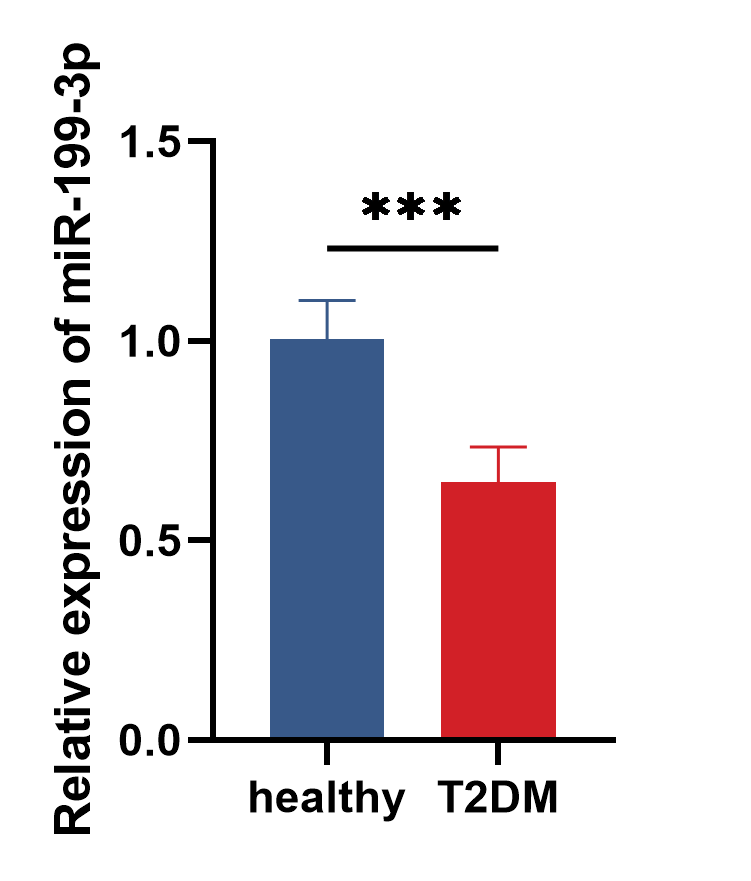

Supplement: Supplementary file 1 — Supplementary Material 1 [file 12872_2023_3601_MOESM1_ESM.png]
